# Myelodysplastic Neoplasms (MDS): Pathogenesis and Therapeutic Prospects

**DOI:** 10.3390/biom15060761

**Published:** 2025-05-25

**Authors:** Xuefeng Li, Chaoyu Zou, Xinrong Xiang, Lei Zhao, Mengran Chen, Chenlu Yang, Yu Wu

**Affiliations:** Department of Hematology, West China Hospital, Sichuan University, Chengdu 610041, China

**Keywords:** myelodysplastic neoplasms, myelodysplastic syndromes, innate immunity, bone marrow microenvironment, gene mutations, therapeutic prospects

## Abstract

Myelodysplastic neoplasms (MDS) are a group of hematological malignancies originating from hematopoietic stem cells (HSCs), characterized by distinct clinical and/or molecular heterogeneity across different MDS subtypes. This review elucidates the pathogenesis of MDS from two main perspectives: the bone marrow microenvironment and recurrent genetic abnormalities. Abnormal bone marrow microenvironment initiates aberrant innate immune response in HSCs, with quantitative and/or functional alterations of immune cells that collectively establish an immunosuppressive microenvironment, and abnormal bone marrow mesenchymal stromal cells that support and promote the progression of MDS. In addition, this review synthesizes current evidence on the biological functions and pathogenic mechanisms of frequently mutated genes in MDS. Furthermore, emerging therapies based on the pathogenesis of MDS are evaluated and summarized. In summary, aberrant innate immune responses promote pyroptosis of HSCs and acquisition of recurrent genetic abnormalities, resulting in the transformation of HSCs into MDS blasts; the immunosuppressive milieu (especially in higher-risk MDS) facilitates immune evasion of MDS blasts, ultimately leading to disease progression. Future research should focus on the interplay between different genetic abnormalities and immune dysregulation, coupled with the development of novel therapies targeting multiple nodes of the pathogenic network, to overcome current challenges in the treatment of MDS.

## 1. Introduction

Myelodysplastic neoplasms (MDS) are a group of hematologic malignancies characterized by distinct clinical and molecular heterogeneity. The classification and risk stratification of MDS have been continuously refined through integration of clinical phenotypes, cytogenetic profiles, and molecular characteristics [1,2]. Epidemiological studies reveal an age-dependent escalation in MDS incidence, which poses heightened therapeutic challenges in clinical management, particularly for elderly patients with compromised baseline health status [3,4]. To improve the therapeutic efficacy and the quality of life in MDS patients, elucidating the molecular mechanisms driving MDS initiation and progression provides a critical foundation for developing mechanism-based interventions.

**Figure 1:**
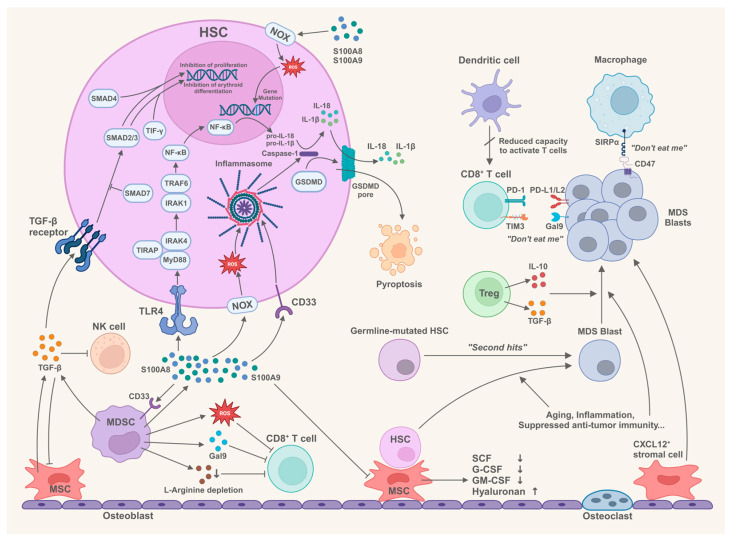
Concise summary for MDS pathogenesis (Created in BioRender. Li, X. (2025) https://BioRender.com/tlr47gv). G-CSF: Granulocyte colony-stimulating factor; GM-CSF: Granulocyte-macrophage colony-stimulating factor; GSDMD: Gasdermin D; HSC: Hematopoietic stem cell; IL: Interleukin; IRAK: Interleukin-1 receptor associated kinase; MDSC: Myeloid-derived suppressor cell; MSC: Mesenchymal stromal cell; MyD88: myeloid differentiation factor 88; NF-κB: Nuclear factor-κB; NK cell: Natural killer cell; NOX: NADPH oxidase; SCF: Stem cell factor; SMAD: Mothers against decapentaplegic homolog; TIRAP: Toll-interleukin 1 receptor domain containing adaptor protein; TLR4: Toll-like receptor 4; TRAF6: Tumor necrosis factor receptor associated factor 6.

The pathogenesis and progression of MDS involve a complex interplay of dysregulated bone marrow microenvironment, the existence of germline mutations that brings the disease susceptibility, and the accumulation of acquired gene abnormalities [5,6]. This review will delineate the mechanistic intricacies of MDS pathogenesis through these dimensions, propose therapeutic prospects based on these mechanisms, and strategically categorize ongoing clinical trials that target these aspects (Figure 1).

## 2. Bone Marrow Microenvironment and the Pathogenesis of MDS

### 2.1. Components in the Bone Marrow Microenvironment Trigger Innate Immune Responses

#### 2.1.1. Innate Immune Signaling in HSCs of MDS

The activation of innate immune responses in HSCs plays a major role in the pathogenesis of MDS, which is triggered by cytokines and inflammatory factors in the bone marrow microenvironment [7]. A previous study reported elevated concentration of interleukin-1β (IL-1β) in the cell culture supernatant of mononuclear cells isolated from the MDS patients’ bone marrow, compared to normal controls (*p* < 0.05), indicating abnormal inflammation in the bone marrow microenvironment of MDS patients [8]. Moreover, Maratheftis et al. demonstrated that the expression of toll-like receptor (TLR)-4 on bone marrow CD34^+^ cells is upregulated in MDS patients compared to those with iron deficiency anemia, underscoring the involvement of innate immune responses mediated by TLRs in the pathogenesis of MDS [9,10]. Subsequent studies further clarified the signal transduction pathways in the innate immune responses of MDS. Damage-associated molecular patterns (DAMPs) or pathogen-associated molecular patterns (PAMPs) in the bone marrow microenvironment bind to TLR-4 on HSCs, activating downstream myeloid differentiation factor 88 (MyD88), and causing the phosphorylation of interleukin-1 receptor associated kinase 4 (IRAK4) and IRAK1. In addition, activated toll-interleukin 1 receptor domain containing adaptor protein (TIRAP) can promote the function of the MyD88/IRAK4 complex. Then, the interaction between phosphorylated IRAK1 and tumor necrosis factor receptor associated factor 6 (TRAF6) serves as a key step in the activation of nuclear factor-κB (NF-κB) pathway [11,12,13,14]. NF-κB is inactive when bound to inhibitor of NF-κB (IκB); however, TRAF6/IRAK1-mediated IκB kinase (IKK) activation triggers the phosphorylation and ubiquitin–proteasome-dependent degradation of IκB, resulting in the dissociation of NF-κB from IκB. The activated NF-κB enters the nucleus and regulates gene expression, initiating transcription of various inflammatory factors or their precursors, e.g., pro-IL-1β and pro-IL-18 [5,11].

#### 2.1.2. S100A8 and S100A9 Drive the Innate Immune Responses in MDS

S100A8 and S100A9 are two members of the S100 protein family, participating in the mechanisms of inflammation and tumorigenesis [15,16]. In MDS, concentrations of S100A8 and S100A9 in bone marrow supernatant exhibit a positive correlation with advancing age, indicating a progressive intensification of age-associated inflammatory burden in the bone marrow [17]. Studies suggested that S100A8 and S100A9 in the bone marrow microenvironment act as DAMPs to initiate the TLR/MyD88/NF-κB signaling axis, which is the central pathway in MDS pathogenesis [7,18]. Additionally, S100A8 and S100A9 also act as ligands to promote the activation of NADPH oxidase (NOX), which exerts dual effects: on the one hand, activated NOX produces reactive oxygen species (ROS) that subsequently activates NOD-, LRR- and pyrin domain-containing protein 3 (NLRP3) and promotes the recruitment and assembly of apoptosis-associated speck-like protein containing a CARD (ASC) and the precursor of Caspase-1 (pro-Caspase-1), ultimately constructing the inflammasome [19,20]. The inflammasome releases biologically active caspase-1, which can turn pro-IL-1β/18 into functional forms. Caspase-1 can also cleave Gasdermin D (GSDMD) to give it the ability to induce the formation of pores in the Fcell membrane. The aforementioned progress will cause pyroptosis of HSCs, and the inflammatory cytokines produced in the process of innate immune responses, such as IL-1β and IL-18, will be released through the pores [21]. The pyroptosis of HSCs induced by innate immune pathways damages the normal hematopoiesis in MDS patients and leads to single- or multiple-lineage refractory cytopenia. On the other hand, ROS conducts the oxidation of nucleoredoxin, resulting in its dissociation from dishevelled. The dissociated dishevelled inhibits the function of β-catenin destruction complex, allowing stable β-catenin to translocate into the nucleus and induce the transcription of cyclin D1 and c-Myc, then promoting the proliferation of HSCs and their transformation to malignant cells [22].

#### 2.1.3. Multifunctionality of MDSCs in the Pathogenesis of MDS

Myeloid-derived suppressor cells (MDSCs) are a group of heterogeneous myeloid cells that can be primarily categorized into two distinct subsets: polymorphonuclear MDSCs (PMN-MDSCs) and monocytic MDSCs (M-MDSCs). The PMN-MDSCs are identified as the expression of CD11b^+^/CD14^−^/CD15^+^ (or CD66b^+^), whereas M-MDSCs express CD11b^+^/CD14^+^/CD15^−^/HLA-DR^-/low^ [23,24]. In MDS, MDSCs are one of the sources of S100A8 and S100A9 in the bone marrow microenvironment; S100A8 and S100A9 secreted by MDSCs bind to the TLRs on the surface of HSCs, and initiate the innate immune responses described above [25,26]. Additionally, elevated expression of CD33 on MDSCs in MDS patients has been observed compared to healthy individuals; the interaction between S100A9 and CD33 on MDSCs facilitates their expansion within the bone marrow, creating a self-perpetuating cycle that exacerbates innate immune responses and drives disease progression [25].

Moreover, activated MDSCs can secrete cytokines such as IL-10 and TGF-β, which have negative effects on hematopoiesis. TGF-β, in particular, is a critical negative regulator of erythroid hematopoiesis [27]. Previous studies have reported that TGF-β inhibits the proliferation of intermediate and late erythroid progenitors while accelerating the differentiation of erythroid progenitors to enucleated erythrocytes [28]. Mechanistically, the activation of TGF-β receptor leads to the formation of complexes such as mothers against decapentaplegic homolog (SMAD)2/3-SMAD4 and SMAD2/3-TIFγ (Transcriptional Intermediary Factor 1γ) in HSCs, resulting in inhibition of HSCs’ proliferation and erythroid differentiation, respectively [29,30,31]. Additionally, SMAD7 can negatively regulate the function of TGF-β receptor I (TBRI), but it is significantly down-expressed in the CD34^+^ cells in MDS, resulting in the enhancement of TGF-β signaling. The inhibitor of TBRI, LY-2157299, has been shown to attenuate TGF-β signaling and stimulate hematopoiesis in primary MDS bone marrow specimens [32]. In addition, Kordasti et al. reported that the serum level of IL-10 in high-risk MDS patients is higher compared to those with low-risk MDS [33]; a meta-analysis also identified that genotype correlated with high expression of IL-10 (−592 CC) is correlated with a lower level of hemoglobin and poorer prognosis [34]. These findings point out that IL-10 may serve as a risk factor in MDS progression, and its secretion by MDSCs underscores their role in MDS pathogenesis.

Furthermore, MDSCs also play a critical role in suppressing immune effects in the bone marrow microenvironment of MDS, facilitating immune evasion of MDS blasts. These mechanisms will be discussed in the subsequent section.

### 2.2. Immune Cells in the Bone Marrow Microenvironment Participate in the Pathogenesis of MDS

#### 2.2.1. Status of CD4^+^ T Cell Subsets in MDS

T cells are a crucial component of human immune system and can be classified into various subsets based on their immunological characteristics as well as pathological and physiological functions, and these subsets play significant roles in the pathogenesis and progression of MDS [35,36]. CD4^+^ T cells are a group of T cells with multipotent differentiation activities. The differentiation of naïve CD4^+^ T cells is regulated by the activation of T cell receptors (TCRs), co-stimulatory receptors, and/or stimulation by different cytokines. After differentiation, distinct CD4^+^ helper T cell (Th) subsets exhibit unique patterns of cytokine secretion and immunoregulatory functions [37,38]. Th1 and Th2 cells are two critical subsets following differentiation of CD4^+^ T cells, and the balance between Th1 and Th2 cells is essential for maintaining immune homeostasis [37]. According to previous studies, the Th1/Th2 ratio in MDS patients compared to healthy controls remains controversial [39,40,41]. One study reported an elevated proportion of Th1 cells in MDS patients, with a positive correlation between Th1 cell proportion and the apoptosis rate of bone marrow nucleated cells [39]. In contrast, another study observed a reduced proportion of Th1 cells in the bone marrow of MDS patients, compared to normal individuals [41]. Therefore, the status of Th1/Th2 balance in MDS still requires further research and validation.

CD4^+^ T cells can differentiate into regulatory T cells (Tregs) upon stimulation by TGF-β, IL-2, TCR/CD28, etc. Tregs play a critical role in maintaining immune tolerance and mitigating immune response [38]. However, inhibitory factors, e.g., TGF-β and IL-10, secreted by Tregs may also contribute to immune evasion and disease progression in MDS [38,42]. Generally, as the risk stratification of MDS increases, the proportion and/or absolute number of Tregs tend to rise. Consequently, a higher proportion of Tregs is often associated with more severe clinical features, such as a higher blasts percentage, a lower hemoglobin level, and poorer overall survival [43,44,45,46].

CD4^+^ T cells can also differentiate to Th9, Th17, Th22, or follicular helper T (Tfh) cells upon other patterns of stimulation [38]. The role of Th17 cells, an IL-17-secreting subset, in MDS remains controversial. One study found that in bone marrow, early-stage (lower-risk) MDS patients exhibit reduced Th17 cell numbers and functional impairment, while later-stage (higher-risk) MDS patients show an increase in Th17 cell numbers [47]. In contrast, other studies reported that in bone marrow and/or peripheral blood mononuclear cells (PBMCs), negative correlations between Th17 cell proportion/number and MDS risk stratification were found, and the lower Th17 cell proportion was associated with more severe clinical features [33,48]. Nevertheless, several studies confirmed that the level of IL-17 in the serum, plasma, or bone marrow of lower-risk MDS patients is elevated compared to those in higher-risk MDS patients, and the higher IL-17 level is correlated with more severe anemia, suggesting the Th17 cells contribute to the pathogenesis of lower-risk MDS [33,48,49]. The role of Th22 cells, which secrete cytokines, such as IL-22 and TNF-α, in MDS has not yet been fully elucidated. Shao et al. observed an expansion of Th22 cells in late-stage MDS patients, accompanied by elevated mRNA expression of TNF-α and IL-6. However, the specific mechanisms underlying the involvement of Th22 cells in MDS pathogenesis remain to be further investigated [50].

#### 2.2.2. Suppression of CD8^+^ T Cells in MDS

CD8^+^ cytotoxic T cells are essential in cellular immunity and tumor surveillance. Previous studies have revealed that the quantity and function of CD8^+^ T cells decline with increasing risk stratification of MDS, reflecting CD8^+^ T cell suppression in the background of disease progression and clonal expansion in MDS; and CD8^+^ T cell suppression will further exacerbate the progression of MDS [46,51,52]. MDSCs play a vital role in the suppression of CD8^+^ T cells, which is partially mediated by the increased production of ROS from MDSCs; high expression of arginase 1 (ARG1) in MDSCs that depletes L-arginine and suppress the proliferation of T cells; and inducible nitric oxide synthase (iNOS) that promotes the production of nitric oxide (NO) and peroxynitrite (ONOO−), then nitrating the T cell receptor (TCR) and disrupting the IL-2 signaling pathway [53,54,55,56,57,58,59]. Furthermore, co-culture of CD8^+^ T cells with Lin^-^/CD33^+^/HLA-DR^-^ MDSCs from MDS patients results in significant reduced proliferation and increased apoptosis in CD8^+^ T cells; the levels of galectin-9 (Gal-9) in serum and bone marrow supernatants from MDS patients, and in culture supernatants of MDS-derived MDSCs, are all higher than those from healthy volunteers; the use of T cell immunoglobulin and mucin domain-containing protein 3/Gal9 (TIM3/Gal9) inhibitors mitigates the CD8^+^ T cells suppression driven by MDSCs, suggesting that in MDS, the TIM3/Gal9 pathway may be the key pathway that contributes to the suppression and exhaustion of CD8^+^ T cells by MDSCs [60]. Additionally, Yu et al. found the TIM3/CEACAM1 (Carcinoembryonic antigen cell adhesion molecule 1) pathway, which is involved in immune checkpoints, correlated with CD8^+^ T cell exhaustion in MDS, and which activates the NF-κB/NLRP3/Caspase-1 pathway in MDSCs, leading to the expression of IL-1β/18 [61]. The STAT3/ARG1 pathway also contributes to the suppression of CD8^+^ T cells induced by MDSCs in MDS; the use of STAT3 inhibitors can partially restore the CD8^+^ T cell function interfered by the MDSCs derived from MDS [62]. Collectively, the suppression and exhaustion of CD8^+^ T cells regulated by MDSCs promote tumor immune escape and compromise the anti-tumor effects in the bone marrow microenvironment [56].

As mentioned above, the exhaustion of CD8^+^ T cells in MDS is associated with the activation of the TIM3 pathway, which provides a rationale for the application of TIM3 inhibitors in higher-risk MDS patients [60,61,63]. Additionally, TIM3 is also upregulated on Tregs and leukemic stem cells/MDS blasts, further supporting the potential therapeutic utility of TIM3 inhibitors in MDS [64,65,66]. Moreover, immune checkpoint molecules such as PD-1, PD-L1, PD-L2, and CTLA4, which are involved in immune evasion from CD8^+^ T cells, are overexpressed in CD34^+^ cells and PBMCs derived from MDS patients. And PD-L1 is also upregulated on tumor-associated MDSCs, which is regulated by the cyclooxygenase-2/microsomal PGE2 synthase 1/prostaglandin E2 (COX2/mPGES1/PGE2) pathway; the blockade of PD-L1 under hypoxic conditions enhances the activation of T cells by reducing IL-6 and IL-10 production from MDSCs [67,68]. These findings highlight the mechanisms of immune evasion in MDS and provide a foundation for the application of PD-1/PD-L1/CTLA4-targeting therapies in MDS [69,70,71].

#### 2.2.3. Dysfunction of NK Cells in MDS

The expression of receptors on NK cells and the levels of their ligands in MDS remain controversial, with conflicting findings reported in the previous studies [72,73]. However, the majority of studies consistently demonstrate that NK cells in MDS patients exhibit functional impairment and abnormalities in maturation, particularly in higher-risk MDS cases [73,74,75]. Upon stimulation with IL-2, MDS-derived NK cells show an increased apoptosis rate and reduced production of immune factors, such as TNF-α and IFN-γ [73]. Moreover, MDS-derived NK cells exhibit significantly decreased expression of DNAM-1, a critical activating receptor for NK cells that plays an essential role in the cytotoxic targeting of MDS blasts [76]. Notably, partial MDS-derived NK cells share the same clonal abnormalities with MDS blasts [73,77]. In MDS patients with TET2 mutations, NK cells were found to harbor the same TET2 abnormalities as MDS blasts. These TET2-mutated NK cells exhibit reduced expression of killer immunoglobulin-like receptors (KIR), perforin, and TNF-α. The downregulation of these effectors is associated with hypermethylation of related pathways and genes. Treatment with hypomethylating agents (HMA) has been shown to effectively restore the phenotype and function of these NK cells. This study provides an additional perspective on the mechanisms underlying the therapeutic effects of HMA in MDS [77]. NK cells can also be influenced by other components within the bone marrow microenvironment. Activation of the TGF-β signaling pathway has been demonstrated to suppress NK cell proliferation, downregulate receptor expression, and impair their anti-tumor activity. Inhibition of the TGF-β pathway enhances the anti-tumor effects of NK cells in various tumor models, including hepatocellular carcinoma and glioblastoma multiforme [78,79,80,81]. Furthermore, inflammatory pressure and accumulated ROS in the MDS bone marrow microenvironment also contribute to the functional impairment and apoptosis of NK cells [82,83,84,85].

#### 2.2.4. Abnormal Macrophages Contribute to the Immune Invasion in MDS

Macrophages represent a group of innate immune cells that activated macrophages can be mainly divided into two categories, M1 and M2 macrophages, based on their functional and phenotypic characteristics [86,87,88]. In various solid tumors, partial macrophages play an important role in the disease progression and are commonly defined as “tumor-associated macrophages” (TAMs) [89,90]. While TAMs in solid tumors predominantly exhibit M2-like phenotypes, in acute myeloid leukemia (AML), the leukemia-associated macrophages (LAMs) display distinct phenotypic profiles. Previous studies suggested that in MLL-AF9-induced murine AML models, bone marrow-derived LAMs tend to exhibit an M1-like phenotype, whereas splenic LAMs are more inclined toward an M2-like phenotype. Importantly, repolarization of LAMs toward the M1 phenotype has been shown to improve survival in AML mouse models [91,92].

In MDS, Yang et al. differentiated PBMCs from MDS patients and healthy controls into macrophages and conducted comparative analyses. Their findings revealed that MDS-derived macrophages mainly exhibit an M2-like phenotype. These M2-polarized macrophages can be repolarized toward an M1-like phenotype upon stimulation with ferric chloride, and the repolarization is reversible with iron chelators [93]. Furthermore, the proportion of M2-like macrophages is significantly higher in higher-risk MDS compared to lower-risk MDS. Macrophages derived from higher-risk MDS demonstrate reduced support ability for HSCs, with enhanced ability to support malignant cells, compared to those from lower-risk MDS [94,95]. Additionally, MDS-derived macrophages exhibit impaired phagocytic function, along with decreased expressions of CD206 and signal regulatory protein α (SIRPα) compared to healthy controls, while showing elevated secretion of iNOS [96]. The interaction between CD47 and signal regulatory protein α (SIRPα) serves as a critical mechanism that how malignant cells evade phagocytosis by macrophages. In various hematologic malignancies, including AML, acute lymphoblastic leukemia (ALL), and non-Hodgkin lymphoma (NHL), CD47 expression is significantly upregulated. This overexpression facilitates immune evasion through its binding to SIRPα on macrophages [97,98,99]. Similarly, in high-risk MDS with increased blasts, elevated CD47 expression has been observed on MDS blasts compared to healthy controls. This finding provides a strong rationale for the potential application of anti-CD47-based therapies in the treatment of MDS [100].

#### 2.2.5. Dysfunction of Dendritic Cells in MDS

Furthermore, dendritic cells (DCs), which serve as antigen-presenting cells for T cells, also exhibit functional abnormalities in MDS. The frequencies of DCs and slan^+^ monocytes in bone marrow are significantly reduced in MDS compared to those in healthy controls. Notably, cDC2 cells (CD1c^+^ DCs) and slan^+^ monocytes in MDS patients display the recurrent karyotypic abnormalities same to those observed in CD34+ cells. Additionally, MDS-derived cDC2 cells and slan^+^ monocytes demonstrate a markedly reduced capacity to induce the proliferation of both CD4^+^ and CD8^+^ T cells [101]. In another study focusing on MDS with systemic inflammatory or dysimmune diseases, a decline in DC populations was also observed, further underscoring the role of DCs in the pathogenesis of MDS [102].

### 2.3. The Role of Bone Marrow Mesenchymal Stem Cells/Stromal Cells in the Pathogenesis of MDS

#### 2.3.1. Impaired Function of MDS-Derived BM-MSCs to Support Normal Hematopoiesis

Bone marrow mesenchymal stem cells are the source of mesenchymal stromal cells, and the bone marrow mesenchymal stromal cells (BM-MSCs) are integral to the formation and maintenance of the bone marrow microenvironment [103]. The BM-MSCs’ functional assessment typically involves evaluating their morphology, differentiation potential, proliferation capacity, and the ability to support HSCs. Researchers found that BM-MSCs derived from MDS patients exhibit reduced capacities of growth and proliferation, along with premature replicative senescence; MDS-derived BM-MSCs also display reduced osteogenic differentiation and altered expressions of key molecules involved in the interactions between BM-MSCs and HSCs, compared to normal counterparts [104,105,106]. Notably, BM-MSCs in MDS show an impaired ability to support HSCs in normal hematopoiesis. Zhao et al. found significantly lower expression of hematopoietic-supporting cytokines, including stem cell factor (SCF), granulocyte colony-stimulating factor (G-CSF), and granulocyte-macrophage colony-stimulating factor (GM-CSF), in MDS-derived BM-MSCs compared to normal controls [107]. The abnormal premature senescence and altered cell status may contribute to the impaired capacity of MDS-derived BM-MSCs to support HSCs. Studies have revealed that senescence of BM-MSCs in MDS is associated with dysregulation of the PI3K/AKT and WNT signaling pathways [108,109]. Additionally, TGF-β within the bone marrow microenvironment can impair the hematopoietic support capacity and osteogenic differentiation potential of MDS BM-MSCs [110]. Furthermore, the activation of innate immune pathways also participates in BM-MSCs dysfunction in MDS. In low-risk MDS, the NF-κB pathway in BM-MSCs is significantly upregulated, resulting in an increased expression of negative hematopoietic regulators [111]. In addition, senescence of mesenchymal stem cells/stromal cells in MDS may also correlate with S100A9, an important molecule involved in the innate immune pathway. Shi et al. found that exogenous S100A9 induces the cellular senescence of primary mesenchymal stromal cells and human stromal cell line HS-27a; while the inhibition of TLR4, ROS, or IL-1β mitigates the senescence of stromal cells induced by S100A9 [112]. Researchers also found that the expression of *DICER1* is downregulated in MDS BM-MSCs and is associated with senescence and functional impairment of BM-MSCs. Overexpression of *DICER1* effectively releases the senescence of BM-MSCs [105,113].

#### 2.3.2. BM-MSCs Correlate with the Prognosis and Progression of MDS

Research revealed the elevated level of hyaluronan in the bone marrow serum from higher-risk MDS patients compared to healthy controls, and the hyaluronan secreted by MDS-derived BM-MSCs is also higher compared to normal controls, especially in the higher-risk MDS; patients with high hyaluronan also showed shorter overall survival (OS) and leukemia-free survival (LFS). These results indicate that the BM-MSCs correlate with risk stratification and prognosis of MDS and highlight the potential of novel therapies targeting BM-MSCs to improve MDS outcomes [114]. Moreover, BM-MSCs isolated from MDS patients demonstrate global DNA hypermethylation. In a murine model transplanted with human hematopoietic stem/progenitor cells (HSPCs) pre-co-cultured with BM-MSCs derived from either MDS patients or healthy donors, co-culture with MDS-derived BM-MSCs was associated with a significantly higher engraftment failure rate. Following the hypomethylating treatment of MDS-derived BM-MSCs, the murine models exhibited improved engraftment efficiency. Notably, in cases where BM-MSCs were refractory to hypomethylating therapies, the murine models displayed accelerated disease progression [115,116,117]. These findings underscore the multifaceted mechanisms underlying the therapeutic effects of HMA in MDS. Additionally, CXCL12^+^ stromal cells participate in forming the bone marrow niche for MDS CD34^+^ hematopoietic cells and supporting their survival. The density of CXCL12^+^ stromal cells is significantly elevated in the bone marrow of patients with MDS and AML with myelodysplasia-related changes (AML-MRC), compared to controls (no morphologic abnormalities in bone marrow) and AML. Further study revealed that CD34^+^ cells in contact with CXCL12^+^ stromal cells exhibit positive expression of BCL2, and cases with higher CXCL12 expression demonstrate reduced apoptosis of their CD34^+^ cells [118,119].

Furthermore, BM-MSCs in MDS also exhibit immunomodulatory properties and contribute to tumor immune evasion [120,121]. BM-MSCs not only respond to negative regulation by TGF-β but are also capable of producing TGF-β themselves. Studies BM-MSCs derived from high-risk MDS secrete a higher level of TGF-β compared to those from low-risk MDS, thereby inducing the production of regulatory T cells and exerting immunosuppressive effects [122]. Additionally, when co-cultured with monocytes, MDS-derived BM-MSCs can induce monocytes to acquire an MDSCs-like phenotype, which subsequently suppresses the function and proliferation of co-cultured NK cells. Further investigation revealed that this process is mediated by the high expression of ENC1, a ROS regulator, in MDS-derived BM-MSCs [123]. Liu et al. directly observed that the proportion of NK cells in the PBMCs of higher-risk MDS patients is significantly lower compared to lower-risk MDS patients and healthy controls. Additionally, the serum level of IFN-γ is also markedly reduced in MDS patients (especially in higher-risk MDS). MDS-derived NK cells exhibit functional impairment by significantly lower expression of NKG2D and perforin. Subsequent study revealed that MDS-derived NK cells display elevated expression of T cell immunoglobulin and ITIM domain (TIGIT); the interaction between TIGIT and CD155 on BM-MSCs contributes to decreased number and impaired function of NK cells in MDS [124]. Collectively, BM-MSCs can promote the progression of MDS in multiple ways.

### 2.4. Therapeutic Prospects in Targeting the Bone Marrow Microenvironment

**Figure 2:**
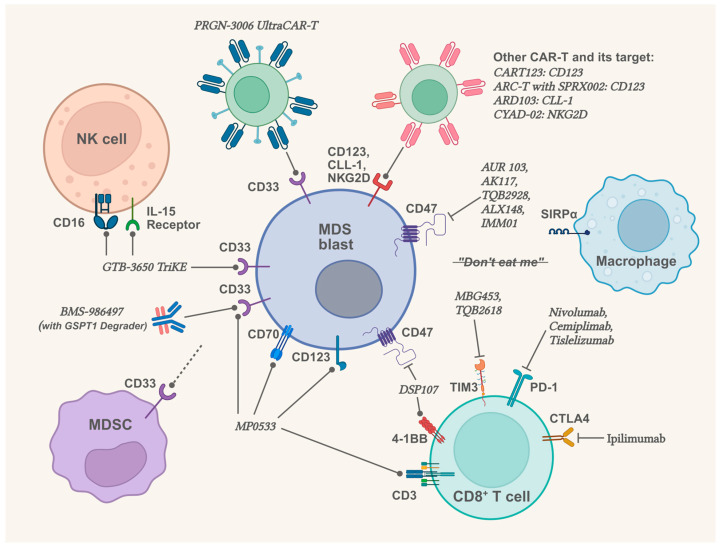
Partial novel therapies under clinical trials for MDS (Created in BioRender. Li, X. (2025) https://BioRender.com/104xdz1). MDSC: Myeloid-derived suppressor cell; NK cell: Natural killer cell.

Therapeutic decision-making in MDS usually requires a comprehensive evaluation of patients’ baseline status, clinical manifestations, risk stratification, and cytogenetic/molecular profiles. For lower-risk patients, guideline-recommended strategies include erythropoiesis-stimulating agents for anemia management, Luspatercept for refractory anemia with SF3B1 mutation, lenalidomide for del(5q) patients with preserved platelet counts, HMAs (azacitidine/decitabine), and supportive cares. Higher-risk MDS management centers on HMAs as first-line therapy, or consideration of AML-like intensive chemotherapy for younger/fit patients, and/or allogeneic hematopoietic stem cell transplantation (allo-HSCT) in eligible candidates with donor availability. For relapsed/refractory MDS, novel agents and clinical trial enrollment are imperative [125,126] (Figure 2).

#### 2.4.1. Therapeutic Prospects Related to Innate Immunity and CD33

Dysregulation of innate immune pathways participates in the pyroptosis of HSCs, recurrent gene mutations, and transformation to more severe hematological malignancies, e.g., AML, in MDS patients. Therefore, targeting aberrant innate immune signaling has emerged as a promising perspective for therapeutic innovation. Canakinumab is a monoclonal antibody targeting IL-1β, which has shown significant efficacy in the treatment of autoimmune diseases, including autoinflammatory recurrent fever syndromes and adult-onset Still’s disease [127,128]. Currently, Canakinumab is being evaluated in several clinical trials for low-risk MDS (NCT04239157, NCT04798339). Another promising therapeutic agent is CA-4948, an inhibitor targeting IRAK4. In a Phase 1 clinical trial, CA-4948 exhibited favorable effectiveness and safety profiles (NCT04278768) (Table 1) [129]. Furthermore, DFV890 is a novel NLRP3 inhibitor that can reduce the production of IL-1β/18; a Phase 1 multi-center clinical trial is ongoing in patients with very low-, low-, or intermediate-risk MDS (NCT05552469).

Since CD33-signaling has important pathogenic effects in MDS, with the elevated expression of CD33 on MDSCs and MDS blasts, CD33 has emerged as a promising therapeutic target [130,131]. BI 836858 is a humanized anti-CD33 antibody with an engineered IgG heavy chain that targets MDSCs expressing a high level of CD33. Researchers found that BI 836858 reduces the number of MDSCs derived from MDS patients through antibody-dependent cellular cytotoxicity (ADCC) and blocks downstream signaling of CD33, resulting in reduced expressions of IL-10 and ROS, genomic stability, and improving the hematopoiesis in low-risk MDS bone marrow specimens ex vivo [132]. However, subsequent Phase 1/2 clinical trials that use BI 836858 to treat low- and intermediate-1-risk MDS patients failed to meet the expected outcomes, indicating that further exploration is needed for treating MDS through CD33/MDSCs (Table 1) [133]. Bispecific or even multi-specific antibodies targeting CD33 may be the solution in future therapeutic development. For instance, GTB-3550 TriKE (Tri-Specific Killer Engager) is a tri-specific drug that binds CD16 and IL-15 on NK cells, CD33 on hematological malignant cells, also using an IL-15 linker to bridge the CD16 and CD33 single-chain variable fragments (scFvs) for sustained cell activation. The result of GTB-3550 TriKE’s Phase 1 study (NCT03214666) demonstrated safety and partial responses in AML and MDS patients [134]. GTB-3650, a next-generation anti-CD16/IL-15/anti-CD33 TriKE, is also under clinical investigation (NCT06594445). Furthermore, MP0533, a tetra-specific CD3-engaging designed ankyrin repeat protein (DARPin) which can target CD33, CD123, and CD70, and induce the death of AML blasts and leukemic stem cells via an avidity-driven T cell-mediated process. Researchers acquired encouraging primary results in both safety and effectiveness, the clinical trial is still ongoing for refractory/relapse AML and MDS/AML patients (NCT05673057) [135]. PRGN-3006 UltraCAR-T, a chimeric antigen receptor (CAR) T cell therapy targeting CD33, achieved encouraging responses in AML participants, and it is still being evaluated in higher-risk MDS participants (NCT03927261) [136]. BMS-986497 (ORM-6151), a targeted protein degrader (TPD) that binds CD33 and releases the degrader of GSPT1 (GSPT1 controls protein translation and is often dysregulated in malignant cells [137,138]), which leads to the death of malignant cells. BMS-986497 is under a Phase 1 study in refractory/relapsed AML and MDS (NCT06419634).

**Table 1:** Clinical trials with results related to innate immunity and CD33.

Drug	Mechanism	Phase	Results or Interim Reports	Register No. or Reference
CA-4948	Inhibitor of IRAK4	Phase 1	All (3 of 3) patients (higher-risk MDS or AML) with spliceosome mutations achieved a marrow CR or better.	NCT04278768 [129]
BI 836858	Inhibitor of CD33	Phase 1/2	Failed to meet expected outcomes in low- and intermediate-1-risk MDS patients.	NCT02240706 [133]
GTB-3550 TriKE	Tri-specific drug (CD33 x CD16 x IL-15)	Phase 1/2	3 of 11 patients (higher-risk MDS or AML) had blast cell decreases, with dose-dependent NK cell activity.	NCT03214666 [134]

#### 2.4.2. Therapeutic Prospects Related to Immune Abnormality in Bone Marrow Microenvironment

As the activation of the TIM3 pathway or immune checkpoint pathways will lead to a “don’t eat me” effect between CD8^+^ T cells and MDS blasts, therapeutic strategies targeting these signaling pathways have emerged to block the immunosuppressive crosstalk. At present, several clinical trials evaluating the use of TIM3 inhibitors are ongoing (NCT04823624, NCT05426798). Sabatolimab (MBG453), a TIM3 monoclonal antibody, demonstrated favorable efficacy and safety when combined with HMA in patients with high-risk or very-high-risk MDS (NCT03066648) [139]. However, when compared to a placebo plus HMA group, the group of Sabatolimab plus HMA, while not increasing the rate of severe adverse events, failed to significantly improve the outcomes of higher-risk MDS patients (NCT03946670) (Table 2). This poses a challenge to the application of TIM3 inhibitors in the treatment of MDS. Therapeutic agents targeting immune checkpoints, including PD-1, PD-L1, and CTLA4, are also undergoing clinical trials in MDS. Pembrolizumab, a humanized PD-1 monoclonal antibody, showed effectiveness when combined with azacitidine in untreated intermediate-1 or higher-risk MDS patients. However, for patients who failed prior HMA therapy, the OR rate was only 25%, and the CR rate was as low as 5%, raising concerns about the efficacy of PD-1-targeting agents in MDS (NCT03094637) (Table 2) [140]. Other PD-1 monoclonal antibodies, Nivolumab, Cemiplimab, and Tislelizumab, as well as the CTLA4 monoclonal antibody Ipilimumab, are also being evaluated in several clinical trials for MDS (NCT02530463, NCT03017820, NCT06536959, NCT02890329). These studies will elucidate the efficacy and safety of immune checkpoint inhibitors in the treatment of MDS. In the field of CAR-T therapy for MDS, in addition to the previously mentioned CD33 target, clinical trials focusing on other targets such as C-type lectin-like molecule-1 (CLL-1), natural killer group 2D (NKG2D), and CD123 are also currently ongoing (NCT06765876, NCT05457010, NCT06680752, NCT04167696).

Given that NK cells in MDS are susceptible to the TGF-β-mediated suppression while also being responsive to the IL-2 activation, a clinical trial combining NK cells, IL-2, and the TGF-β inhibitor Vactosertib is currently being conducted across various malignancies, including MDS (NCT05400122). IL-15, another activator of NK cells, has a potential advantage over IL-2 by possibly avoiding the co-activation of Tregs [72,141,142]. Several clinical trials have explored the use of IL-15/IL-15 receptor agonists in combination with NK cell infusion for the treatment of AML. However, the combination of NK cells with IL-15 receptor agonists does not significantly improve clinical outcomes compared to IL-2-based approaches. This may be attributed to the enhanced IL-15 signaling, which promotes the function and proliferation of CD8^+^ T cells, thereby exacerbating the rejection and clearance of allogeneic NK cells (NCT01898793) [143]. Consequently, the therapeutic potential of IL-15 combined with NK cell therapy for hematologic malignancies remains uncertain.

Significant progress has been made in the development of CD47-based therapeutic approaches for MDS [144,145]. Researchers found that treatment with azacitidine leads to a 4- to 6-fold upregulation of CD47 expression in MDS cell lines (MOLM-13 and SKM-1), which may enhance the efficacy of anti-CD47 monoclonal antibodies [146]. However, despite a phase 1b clinical trial combining azacitidine with the anti-CD47 antibody magrolimab demonstrated encouraging results in higher-risk MDS patients, the subsequent phase 3 clinical trial failed to show superior efficacy of the azacitidine + magrolimab combination compared to azacitidine + placebo. Moreover, the addition of magrolimab is associated with a higher incidence of severe adverse events. Consequently, clinical trials evaluating magrolimab in higher-risk MDS have been discontinued (NCT03248479, NCT04313881) (Table 2) [147]. Currently, several anti-CD47 monoclonal antibodies are still receiving evaluations of their efficacy and safety in treating MDS (NCT05607199, NCT04900350, NCT06008405). Additionally, a next-generation CD47 antagonist, ALX148 (Evorpacept), consisting of two engineered high-affinity CD47-binding domains of SIRPα linked to an inactive Fc domain, has been developed. This design confers significantly higher affinity for CD47 compared to native SIRPα on macrophages, enabling more effective blockade of CD47/SIRPα signaling. Promising efficacy and safety data have been reported from a phase 1b clinical trial evaluating ALX148 in combination with azacitidine in higher-risk MDS patients (NCT04417517) (Table 2) [148]. Furthermore, IMM01, a compound with a similar mechanism to ALX148, also demonstrated encouraging preliminary results in its phase 2 clinical trial and is currently under further investigation (NCT05140811) (Table 2) [149]. Moreover, CD47/SIRPα-targeted bispecific antibodies (BsAbs) represent a promising direction for therapeutic development. Given the critical role of CD33 in the innate immune response of MDS, CD47/CD33 BsAbs hold potential for clinical efficacy. HMBD004, a bispecific antibody targeting both CD47 and CD33, has demonstrated the ability to prolong progression-free survival (PFS) in murine models of AML. However, its efficacy in MDS remains to be evaluated [150]. Additionally, 4-1BB (CD137) is a co-stimulatory receptor whose activation can enhance the anti-tumor/infection functions of both T cells and NK cells [151,152]. The CD47/4-1BB bispecific antibody DSP107 is currently under clinical investigation for MDS (NCT04937166).

**Table 2:** Clinical trials with results related to immune abnormalities in the bone marrow microenvironment.

Drug	Mechanism	Phase	Results or Interim Reports	Register No. or Reference
Sabatolimab	TIM3 monoclonal antibody	Phase 2	Sabatolimab plus HMA failed to meet the primary efficacy objectives in higher-risk MDS patients compared to placebo plus HMA (CR: 21.5% vs. 17.7%; median PFS: 11.07 vs. 8.48 months; both *p* > 0.05).	NCT03946670
Pembrolizumab	PD-1 monoclonal antibody	Phase 2	For untreated higher-risk MDS patients, Pembrolizumab plus azacitidine reached the OR rate of 76% and the CR rate of 18%; for patients failed prior HMA therapy, the OR rate was only 25%, and the CR rate was only 5%.	NCT03094637, [140]
Magrolimab	CD47 monoclonal antibody	Phase 3	In untreated MDS patients, azacitidine plus magrolimab showed a lower CR rate and shorter OS compared to azacitidine plus placebo (CR: 21.3% vs. 23.6%; median OS: 15.9 vs. 18.6 months).	NCT03248479, NCT04313881, [147]
ALX148 (Evorpacept)	CD47-blocking fusion protein	Phase 1b	ALX148 plus azacitidine: in 5 newly diagnosed higher-risk MDS patients (all had *TP53* mutation), 1 reached marrow CR, 2 reached cytogenetic response; in 5 relapsed/refractory MDS patients, 2 reached marrow CR.	NCT04417517, [148]
IMM01	CD47-blocking fusion protein	Phase 2	In 17 higher-risk MDS patients who received IMM01 plus azacitidine for ≥6 months, the OR rate was 88.2%, and the CR rate was 41.2%.	NCT05140811, [149]

#### 2.4.3. Therapeutic Prospects Related to BM-MSCs

The studies on abnormal bone marrow mesenchymal stem cells/stromal cells in MDS also provide therapeutic perspectives for MDS. Boada et al. reported that azacitidine can reduce the production of inflammatory factors (i.e., IL-6) by BM-MSCs, suggesting that azacitidine has another mechanism in treating MDS [153]. In addition, TGF-β secreted by MDSCs not only directly interferes with erythropoiesis but also correlates with abnormalities in mesenchymal stem cells/stromal cells. Geyh et al. identified TGF-β1 signaling as a common cause of gene expressions in AML- and MDS-derived BM-MSCs; TGF-β1 can induce dysfunction of healthy BM-MSCs and damage their hematopoietic support ability. SD-208, an inhibitor of TGF-β receptor signaling, restores the osteogenic differentiation and hematopoietic support capacities of AML/MDS-derived BM-MSCs, indicating a therapeutic potential for the future treatment of MDS [110]. Elritercept (KER-050), a novel inhibitor of TGF-β signaling, achieved durable transfusion independence in IPSS-R very low-, low-, or intermediate-risk MDS patients according to its Phase 2 results [154]. A Phase 3 double-blind study is further evaluating its efficacy and safety in lower-risk MDS with anemia (NCT06499285).

## 3. Recurrent Gene Abnormalities in the Pathogenesis of MDS

### 3.1. Pathogenic Mechanisms of Recurrent Gene Abnormalities in MDS

Recurrent gene abnormalities are frequently observed in MDS patients and have been properly summarized in previous studies [6,155]. This section will systematically review the most frequent mutations in MDS, focusing on the latest research findings of their pathogenic mechanisms in MDS, and summarize current clinical trials targeting these genetic aberrations (Table 3, Table 4 and Table 5).

#### 3.1.1. Cohesin Complex Member STAG2 in MDS

*STAG2* mutations are identified in 4–10% of MDS patients and are associated with significantly worse median survival and OS [166,205]. Loss of *Stag2* leads to reduced chromatin accessibility and transcription of lineage-specification genes, enhancing HSCs’ self-renewal, impairing differentiation, and promoting myeloid dysplasia [206]. Tothova et al. developed a murine model with mutant *Tet2* or co-mutant *Tet2/Stag2*. Compared to Tet2-mutant mice, the co-mutant *Tet2/Stag2* mice exhibit more severe phenotypes, including leukocytosis, anemia, and thrombocytopenia. The co-mutant *Tet2/Stag2* bone marrow cells also display higher levels of double-strand DNA breaks and sensitivity to talazoparib, an inhibitor of poly ADP-ribose polymerase (PARP) to suppress the DNA damage repair, in vitro [207]. Another study found that the acquisition of *STAG2* mutant clones makes TNFα-induced pro-survival NF-κB signaling become the major pathway for MDS HSCs’ survival, rather than BCL2-mediated anti-apoptotic pathways, resulting in resistance to venetoclax [208].

#### 3.1.2. RAS Signaling-Related Genes in MDS

Mutant *NRAS* is one of the risk factors for MDS patients transforming to AML, highlighting the adverse effects of aberrant RAS signaling in the progression of MDS [209,210]. Ren et al. reported that MDS patients with mutations in the RAS pathway (including *NRAS*, *KRAS*, *CBL*, *PTPN11*, and *NF1*) demonstrate a higher IPSS-R classification, a shorter OS, and a higher rate of AML transformation [211]. Notably, *NRAS* and *PTPN11* mutations are more prevalent in secondary AML than in higher-risk MDS, and the *KRAS* mutation is more frequent in higher-risk MDS than in lower-risk MDS, implying that RAS signaling abnormalities tend to occur in the late stage of MDS [187]. In addition, the *KRAS^G12D^* mutation in non-hematopoietic cells within the bone marrow microenvironment induces MDS phenotypes in murine models, accompanied by the upregulation of IL1-superfamily members and the NLPR3 inflammasome [212]. However, the precise mechanisms by which RAS signaling contributes to MDS initiation and progression remain to be fully elucidated.

#### 3.1.3. TP53 Abnormalities in MDS

*TP53* encodes the tumor suppressor p53 and is one of the most commonly mutated genes in malignancies. In MDS, the presence of multiple *TP53* hits (including multiple mutations, mutation(s) with deletion, or mutation(s) with copy-neutral loss of heterozygosity) predicts a higher risk of transformation into AML, a poorer response to traditional treatments, and inferior survival outcomes [213,214]. P53 is essential for maintaining hematopoietic homeostasis, and its dysfunction plays an early role in initiating the formation of premalignant HSCs and promoting the progression to hematological malignancies [215,216]. Sallman et al. demonstrated that MDS and secondary AML patients with *TP53* mutations exhibit elevated PD-L1 expression in HSCs, which is driven by the upregulation of *MYC* and the downregulation of MYC’s negative regulator *miR-34a*. Additionally, the numbers of OX40^+^ cytotoxic T cells, helper T cells, and ICOS^+^/4-1BB^+^ NK cells in the bone marrow of patients with *TP53* mutations significantly reduce, alongside increased immunosuppressive regulatory T cells and MDSCs. These findings indicate profoundly altered bone marrow microenvironment and immune environment in patients with *TP53* mutations [217]. In addition, haploinsufficiency of del(5q) genes with *Tp53* loss can induce AML in murine models; and the loss of 5q with *TP53* mutations promotes the structural and karyotypic abnormalities in isogenic MDS induced pluripotent stem cells (iPSC) by perturbing genome stability [218,219].

#### 3.1.4. Germline Alterations in MDS

Germline alterations are increasingly recognized as contributors to the disease susceptibility of MDS, particularly in pediatric and younger adult patients. Studies report that 13.6% to 22.6% of MDS patients harbor germline pathogenic alterations [220,221,222]. *DDX41* is one of the most common germline alterations in adult MDS. Chlon et al. reported that *DDX41* monoallelic mutations confer a competitive advantage to HSPCs, and mice with *Ddx41* monoallelic mutations exhibit age-dependent hematopoietic defects, which is similar to the characteristics of human MDS; the biallelic *DDX41* alterations (germline plus somatic mutation) cause ribosome defects and reduced translation of protein, leading to apoptosis and myelodysplasia of the HPC [223]. In addition, Weinreb et al. found that mutant *DDX41* results in the accumulation of R-loops, activated inflammatory pathways, and increased HSPCs production, providing additional insights into the mechanisms of *DDX41*-driven hematopoiesis dysregulation [224]. Nagata et al. reported that loss-of-function *SAMD9/SAMD9L* germline alterations lead to increased proliferation of HSPCs, while gain-of-function germline alterations of *SAMD9/SAMD9L* cause reduced proliferation. The second hits (i.e., somatic mutations or abnormal karyotypes) will promote the HSPCs harboring germline *SAMD9/SAMD9L* alterations to MDS [225]. Similarly, the malignant transformation initiates when germline *GATA2* deficiency harbors random loss of chromosome 7/7q and receives the second MDS-related somatic mutations [226,227]. Conclusively, these findings underscore the need for further exploration of germline alterations and their interactions with acquired mutations in MDS pathogenesis.

### 3.2. Therapeutic Prospects for Gene Abnormalities in MDS

Given the prevalence of recurrent gene abnormalities in MDS, numerous targeted therapies are under development. These therapies aim to inhibit mutant proteins, modulate upstream or downstream signaling pathways, or restore normal protein function, etc. Combining novel targeted agents with existing regimens (i.e., hypomethylating agents) is also one of the directions for future treatment of MDS. Table 6 presents the current drug development and clinical trials related to gene abnormalities in MDS.

## 4. Discussion and Conclusions

Nowadays, the clinical management and development of novel agents in MDS remain challenging. Advances in the field of pathogenic mechanisms of MDS enable the application of related inflammatory factors of cytokines for clinical work. For instance, elevated concentrations of S100A8 and/or S100A9—key initiators of abnormal innate immunity in MDS—have shown effectiveness in the differential diagnosis between MDS and aplastic anemia; and their heterodimer’s level may also serve as a prognostic biomarker for MDS [17,228,229]. The other components in the pathogenesis of MDS, such as hyaluronan, TGF-β, IL-10, etc., may also have potential value in differential diagnosis, risk stratification, prediction for treatment response, and/or prediction for prognosis in MDS [230,231,232]. Further studies are needed to explore or validate their values in the clinical management of MDS (Table 7).

Since a dysregulated bone marrow microenvironment can initiate the pyroptosis and transformation of HSCs, promote the acquisition of genetic/karyotypic abnormalities, and demonstrate a suppressed anti-tumor immunity, amounts of novel therapies based on these mechanisms are under clinical trials. However, the primary results of several novel agents (relating to CD33, PD-1, TIM-3, and CD47) were suboptimal or nonsignificant (NCT02240706, NCT03946670, NCT03094637, NCT04313881). This limited therapeutic response may correlate with two aspects. On the one hand, the pathological microenvironment in MDS involves intricate regulatory networks and cellular crosstalk, which may compensate or resist an intervention targeting a single mechanism. On the other hand, the mechanistic correlations between karyotypic/genetic alterations and immune dysregulation in the microenvironment remain to be further explored, and these novel therapies may not overcome the pathogenic effects brought by existing chromosomal/genetic alterations. Therefore, developing multi-target drugs or using combination therapies that target multiple pathways may lead to better clinical outcomes. Furthermore, current emerging therapies mainly focus on higher-risk MDS patients. However, the more dominant immunosuppression in the bone marrow microenvironment of higher-risk MDS (i.e., with *TP53* mutations) may limit the effectiveness of these treatments [46,74,94,234]. Therefore, future clinical trials should more adequately classify participants based on certain karyotypic/genetic characteristics; furthermore, whether these novel immunotherapies show greater efficacy in lower-risk MDS needs to be addressed and discussed.

In the future, advances in understanding the molecular pathogenesis of MDS will provide new perspectives for therapeutic development. Novel therapies targeting the underlying pathogenic mechanisms of MDS hold promise for improving both survival outcomes and quality of life in patients with this disease.

## Figures and Tables

**Table 3:** Chromatin modification and transcription factor genes in MDS.

Gene	Wide-Type’s Main Biological Functions	Mutational Frequencies and Clinical Significance	Mechanisms of Mutational Effects
*ASXL1*	Cooperates with PRC2 to facilitate H3K27me3; cooperates with PRC1 to facilitate H2AK119Ub; downregulates the *HOXA* cluster via PRC2. The OGT-ASXL1 axis mediates methylation of H3K4 [156,157,158,159].	15–20%. Some studies report poorer OS in *ASXL1*-mutated MDS patients [160,161].	*ASXL1* mutation or knockdown causes loss of PRC2-mediated H3K27me3 and upregulation of *HOXA* genes, leading to impaired hematopoiesis and MDS-like phenotypes [158,162]. *ASXL1* mutation reduces the expression of genes related to erythroid differentiation and/or maturation, and reduction in H2AK119Ub is related to leukemic transformation [158,162,163].
*EZH2*	Catalyzes the recruitment of PRC2 by interacting with ASXL1; maintains normal H3K27me3 levels [158,164].	5–10%. *EZH2* mutations often co-mutate with *TET2* or *RUNX1*; but the prognostic values of mutant-*EZH2* remain uncertain [165,166,167].	In MDS, loss-of-function *EZH2* mutations are more frequent [164]. Loss of *EZH2* function with *TET2^KD/KD^* induces aberrant DNA hypermethylation and promotes the pathogenesis of MDS [168,169].
*RUNX1*	Key transcription factor for hematopoiesis. Involved in the epigenetic regulation [170,171].	10–15%. Mutant-*RUNX1* correlates with worse OS and LFS in MDS [172].	*RUNX1* mutation with deletion of miR-146a can drive the transformation of normal HSPCs to MDS, and subsequent progression to AML [173]. *RUNX1* mutations lead to the elimination of the DDR-mediated senescence barrier and promote the progression of MDS [174]. *RUNX1*-mutated HPCs from edited Fanconi anemia iPSC have higher expression of IRAK1 and activated NF-κB pathway and show MDS-like phenotypes [175]. *RUNX1* deficiency with *SRSF2* mutation induces MDS phenotype by causing mis-splicing of genes in the DDR and cell cycle checkpoint pathways [176]. Co-deficiency of STAG2/RUNX1 induces MDS-like phenotypes by disrupting enhancer-promoter looping dynamics and downregulating genes with high basal transcriptional pausing [177].

CHIP: clonal hematopoiesis of indeterminate potential; DDR: DNA damage response; H2AK119Ub: ubiquitination of histone H2A at lysine 119; H3K27me3: trimethylation of histone H3 lysine 27; HOXA: homeobox A; HPCs: hematopoietic progenitor cells; HSPCs: hematopoietic stem and progenitor cells. iPSC: induced pluripotent stem cell; LFS: leukemia-free survival; MPN: myeloproliferative neoplasms; OGT: O-linked N-acetylglucosamine transferase; OS: overall survival; PRC: polycomb repressive complex.

**Table 4:** DNA methylation-related genes in MDS.

Gene	Wide-Type’s Main Biological Functions	Mutational Frequencies and Clinical Significance	Mechanisms of Mutational Effects
*DNMT3A*	Encoding enzymes for initiating de novo DNA methylation, catalyzing the conversion of unmethylated cytosine to methylated status at CpG sites [6,155].	10–15%. *DNMT3A* mutation is an independent risk factor for death in patients with MDS [178].	*Dnmt3a*-KO mice show MDS-like phenotypes and hepatomegaly. The *Dnmt3a*-null progenitor cells show global hypomethylation and reactivation of fetal liver hematopoiesis transcriptional programs [179].
*TET2*	Cooperates with α-KG to demethylate DNA by hydroxylating 5-methylcytosine [6].	20–30%. The prognostic value of *TET2* mutations remains uncertain [178,180].	The absence of TET2 leads to increased expression of IL-6 and IL-1β in response to inflammatory stimuli, enhancing innate immune responses in MDS [181,182]. Reduced expression of SIRT1 in MDS HSPCs leads to *TET2* hyperacetylation, enhanced self-renewal and maintenance of MDS HSPCs [183]. *TET2* deletion in MDS HSPCs results in a reduced global level of 5hmC; the deficiency of *TET2* activity increases the risk of MDS transforming to AML by a higher occurrence of secondary malignant mutations [184].
*IDH1* or *IDH2*	Converts isocitrate to α-KG; α-KG with TET2 hydroxylates 5-methylcytosine [185,186].	2–5%. *IDH2* mutations are more prevalent in high-risk MDS than low-risk MDS [187].	Abnormal IDH encoded by mutant *IDH1/2* catalyzes α-KG to R-2-HG, which promotes the occurrence and progression of AML by reducing global levels of 5hmC and inhibiting KDM5 histone lysine demethylases [188]. In murine models, R-2-HG inhibits oxoglutarate dehydrogenase activity and leads to reduced production of CoA, then the insufficiency of succinyl-CoA attenuates the biosynthesis of heme in *IDH1*-mutant hematopoietic cells and induces abnormal erythropoiesis [189].

5hmC: 5-hydroxymethylcytosine; α-KG: α-ketoglutarate; CoA: Coenzyme A; KDM5: Lysine demethylase 5; R-2-HG: D/R-2-hydroxyglutarate.

**Table 5:** RNA splicing-related genes in MDS.

Gene	Wide-Type’s Main Biological Functions	Mutational Frequencies and Clinical Significance	Mechanisms of Mutational Effects
*SF3B1*	SF3B1 is the core component of the spliceosome [190].	20–30%. Notably, *SF3B1* mutations are more frequent in patients with MDS with ring sideroblasts and are associated with a relatively better prognosis [166,191]. However, *SF3B1^K666N^* mutation may correlate with poorer prognosis compared to other *SF3B1* mutations in MDS patients [192].	Mutated *SF3B1* induces aberrant splicing of *IRAK4*, resulting in a long IRAK isoform that leads to hyperactivation of the NF-κB pathway [193]. Mutated SF3B1 induces aberrant splicing of the iron transporter *ABCB7*, leading to reduced *ABCB7* expression and iron accumulation in the mitochondria in erythroid progenitors [194]. Mis-splicing of *ERFE*, a key regulator of iron homeostasis, further exacerbates iron dysregulation in *SF3B1*-mutant MDS [195]. Mutated SF3B1 induces the accumulation of R-loops in MDS and leukemia cells, contributing to DNA damage and genomic instability [196].
*SRSF2*	Regulates pre-mRNA splicing in the nucleus [197].	10–15%. *SRSF2* mutations often co-mutate with *IDH2* mutations and are associated with a shorter leukemia-free survival in MDS [167].	Conditional expression of the *SRSF2^P95H^* mutation in murine models recapitulates MDS phenotypes, driven by mutant SRSF2’s altered preference for specific exonic splicing enhancer motifs [198]. The mis-splicing results in the aberrant isoforms of some key hematopoietic regulators and degradation of the EZH2, impairing hematopoietic differentiation and increasing leukemic risk [198,199]. *SRSF2^P95H/+^* impairs the splicing of mitochondrial mRNAs, increases mitophagy, and elevates the expression of *PINK1* (which is vital for the survival of *SRSF2*-mutant cells) [200].
*U2AF1*	In pre-mRNA splicing, U2AF1 participates in the recognition of the 3’ splice site, and is essential for the maintenance and normal function of HSPCs [201].	5–10%. MDS patients harboring *U2AF1* mutations generally present with a poorer prognosis [178,202].	Mutant U2AF1 leads to high-activity isoform long IRAK4, amplifying downstream innate immune responses [203]. SKM-1 and K562 cells with *U2AF1^S34F^* mutation show reduced proliferation and increased apoptosis, and the *U2AF1^S34F^* SKM-1 cells show elevated mRNA of *FOXO3a*; the dysregulation of FOXO3a restores autophagy flux and activates the NLRP3 inflammasome [204].

**Table 6:** Ongoing clinical trials focusing on certain gene abnormalities in MDS.

Drugs	Phase	MDS Types and Gene Abnormalities	Main Mechanisms	Register No.
Luspatercept	Phase 2	Lower-risk MDS with splicing mutation (*SRSF2*, *U2AF1*, *ZRSR2*), or with *SF3B1* mutation and received HMA and/or lenalidomide prior treatments	Binding to TGF-β and reducing SMAD2/3	NCT05732961
Emavusertib (CA-4948)	Phase 1/2	Refractory/relapse (R/R) higher-risk MDS with spliceosome mutations of *SF3B1* or *U2AF1*	Inhibitor of IRAK4 and FLT3	NCT04278768
Eltrombopag	Phase 2	Lower-risk MDS with *TET2* mutations	Thrombopoietin receptor agonist and inhibiting the growth of *TET2*-mutated cells	NCT06630221
Ivosidenib-based therapies	-	MDS with *IDH1* mutation (The specific types of MDS depend on the study designs)	Inhibitor of mutant IDH1	NCT02074839; NCT04250051; NCT03471260; NCT03839771
Olutasidenib-based therapies	-	MDS with *IDH1* mutation (The specific types of MDS depend on the study designs)	Inhibitor of mutant IDH1	NCT06543381; NCT06597734
Enasidenib-based therapies	-	MDS with *IDH2* mutation (The specific types of MDS depend on the study designs)	Inhibitor of mutant IDH2	NCT03744390; NCT06577441; NCT03839771;
Oral Arsenic Trioxide	Phase 2	MDS with *TP53* mutation	Rescuing structural p53 mutations	NCT06778187
Quizartinib	Phase 1/2	MDS with *FLT3-ITD* mutation, or presence of *CBL* exon 8 or 9 deletions or point mutations	Inhibitor of FLT3	NCT04493138
Gilteritinib-based therapies	-	MDS with *FLT3* mutations (The specific types of MDS depend on the study designs)	Inhibitor of FLT3	NCT04027309; NCT05010122

**Table 7:** Prognostic correlations of inflammatory factors/cytokines in MDS.

Components	Prognostic Correlations	References
S100A8/A9 heterodimer	High concentration of S100A8/A9 heterodimer in bone marrow plasma (cutoff: 7093 ng/mL) is correlated with worse LFS and OS.	[229]
Hyaluronan	Higher concentration of hyaluronan in bone marrow serum (>100 μg/L) is correlated with worse LFS and OS.	[114]
IL-6, IL-7, and CXCL10	Patients with normal plasma levels of IL-6, IL-7, and CXCL10 have better OS than those with elevated levels of at least one of the three cytokines; elevated level of IL-6 correlates with worse LFS.	[232]
High inflammatory load	High inflammatory load (IL-6, TNF-α, IL-10, and CXCL10) in blood plasma correlates with shorter OS in clonal cytopenias of undetermined significance (CCUS) and MDS.	[233]

## Data Availability

Not applicable.

## Abbreviations

The following abbreviations are used in this manuscript:

AML	Acute myeloid leukemia
BM-MSCs	Bone marrow mesenchymal stromal cells
DAMPs	Damage-associated molecular patterns
HSCs	Hematopoietic stem cells
HSPCs	Hematopoietic stem/progenitor cells
LFS	Leukemia-free survival
MDS	Myelodysplastic neoplasms
MDSCs	Myeloid-derived suppressor cells
OS	Overall survival
ROS	Reactive oxygen species
TLR	Toll-like receptor
